# Epidemiology of Hepatitis Delta Virus infection in HIV-infected individuals in Taiwan

**DOI:** 10.1186/1742-4690-9-S1-P49

**Published:** 2012-05-25

**Authors:** Hsi-Hsun Lin, Susan Shin-Jung Lee, Ming-Lung Yu, Bo-Sean Hu, Shiou-Haur Liang, Wen-Chien Ko, Jaw-Ching Wu, Fan-Ceng Zheng, Chung-Hsu Lai, Jin-Long Lin

**Affiliations:** 1 E-Da Hospital, I-Shou University, Kaohsiung, Taiwan, Province of China

## Introduction

HIV-infected individuals are at higher risk for acquiring HDV. We sought to study the prevalence, genotypes, and associated risk factors causing HDV infection in HIV-infected individuals from an area with high prevalence of hepatitis B virus infection.

## Materials and methods

A multicenter study of 341 (22.1%) HBsAg+ from 1543 HIV-infected patients was conducted from 2005 through 2011. Blood samples were collected and analyzed for the presence of antibody to HDV and to determine the genotype of HDV.

## Results

The overall prevalence of HDV infection among HBsAg+ carriers was 54.8% (187/341). However, the prevalence among different risk group was distinct. The prevalence of HDV was 73.6%, 13.5%, and 9.2% among HIV-infected IDUs, heterosexual, and MSM, respectively. The main circulating HDV subtypes in our study were genotype IV (60.5%), genotype II (27.6%), and genotype I (11.8%). Multivariate logistic regression analysis revealed that the major risk factor associated with HDV infection was injection drug use, following by HCV infection, HBsAg titer >=250 IU/mL, and duration of injection drug use. A significant increase of cumulative seroprevalence of HDV with duration of IDU from 1 to 15 years was observed (OR: 1.20, 95% CI: 1.09-1.32, P<0.01).

## Conclusions

Our study demonstrated high prevalence of HDV infection among HIV-infected IDUs. Effective strategies are needed to prevent injection drug use and to educate ongoing IDUs about the avoidance of practices that lead to infection with HIV, HCV, and HDV.

